# Supplementary material for: Comparison of ChatGPT-4o and expert evaluation in endodontic education: a cross-sectional pilot study
Source: BMC Med Educ. 2025 Dec 1;26:12. doi: 10.1186/s12909-025-08358-2 (PMC12777446; doi:10.1186/s12909-025-08358-2)
Supplement: Supplementary file 2 — Supplementary Material 2. [file 12909_2025_8358_MOESM2_ESM.docx]

**Comparison of ChatGPT-4o and Expert Evaluation in Endodontic Education**

**Authors**Suha Alpay^1^ ,Yasemen Darafarin^1^ ,Burcu Dagdelen^1^ ,Sana Mahroos Mkhailef Al-Shammari^1^ ,Isil Kaya Buyukbayram^1^

^1^Department of Endodontics, Faculty of Dentistry, Istanbul Aydın University, Istanbul, Türkiye

salpay@aydin.edu.tr, darafarin.yasemen@gmail.com, burcudagdelen@stu.aydin.edu.tr, sanamahroosmkhailefa@aydin.edu.tr, isilkayabuyukbayram@aydin.edu.tr

**Corresponding author**Suha Alpay, salpay@aydin.edu.tr
Department of Endodontics, Faculty of Dentistry, Istanbul Aydın University, Istanbul, Türkiye

**Abstract**

**Background:**
Artificial intelligence (AI) has the potential to enhance objectivity and scalability in educational assessment, yet its role in evaluating technical dental skills remains unclear. This study aimed to compare ChatGPT-4o based assessments with expert evaluations in undergraduate endodontic training and to explore student perceptions of AI-assisted feedback.

**Methods:**
This cross-sectional pilot study was conducted during the 2024–2025 academic year with 32 dental students from a faculty of dentistry, who completed root canal treatments. Postoperative radiographs were evaluated by 10 years experienced endodontist and ChatGPT-4o was used to evaluate performance based on five standardized criteria: canal centering, homogeneity, procedural errors, apical shaping, and overall taper, each rated on a 5-point Likert scale. Inter-rater reliability was assessed via intraclass correlation coefficients (ICC), and Pearson correlation tested linear alignment. Students rated both feedback sources via Likert-scale questionnaires and open-ended comments; paired-sample t-tests compared the mean scores.

**Results:**
Agreement between AI and expert evaluation was limited, with ICC ranging from 0.36 to 0.45, indicating poor to moderate reliability. Pearson r values were <0.3 and not statistically significant, demonstrating weak linear correlation. While students rated AI-generated feedback as moderately useful, expert feedback scored higher across educational value (mean 4.29 vs. 3.90), clinical reasoning support (4.19 vs. 4.06), and reliability (4.00 vs. 3.91); differences were not statistically significant. Notably, 53.1% of students preferred a combination of AI and expert feedback for optimal learning.

**Conclusions:**
AI-generated feedback was moderately useful to students, but expert feedback consistently scored higher. For complex psychomotor skills and radiographic interpretation, AI should serve as an auxiliary tool rather than an independent assessor. Further validation of advanced multimodal AI systems and development of hybrid frameworks combining algorithmic objectivity with expert judgment are recommended.

**Trial registration:**
Not applicable.

**Keywords:** *artificial intelligence, chatgpt-4, endodontic education, dental education*

**1. Background**

The field of dentistry stands to benefit significantly from the potential of artificial intelligence (AI) to transform various aspects of the profession, including diagnostics, prognostics, telemedicine, treatment workflows, operational efficiency, research, and education [1]. As its integration into healthcare education expands, AI is reshaping traditional teaching and assessment paradigms. In addition to conventional instructor-led methods, students now engage with AI-based educational tools that simulate human decision-making processes, thereby enhancing their knowledge and clinical reasoning skills [2]. To connect theory with practice, we should examine how general AI capabilities can be used to standardise approaches to diagnosis and treatment. Standardising approaches may improve consistency and equity in education and clinical environments [3].

Large language models (LLMs), for instance OpenAI's ChatGPT-4, have exhibited remarkable proficiency in generating clinically coherent responses and demonstrating excellence in medical examinations, including the USMLE [4]. LLMs are still in the early stages of use in dentistry. Endodontics involves manual skills, anatomical knowledge and procedural accuracy, usually evaluated during preclinical training [1]. These practical assessments are imperative for clinical competence, yet substantial variability has been observed in expert evaluations, even when structured assessment are employed [5]. Despite the continuous development and refinement of AI technologies, which offer scalable, consistent, and instantaneous feedback, the prevailing approach in a variety of contexts, including high-stakes or skill-based assessments, remains expert evaluation. This tradition is particularly robust in domains where accuracy and human expertise are paramount.

A key part of endodontic training, root canal shaping involves multiple measurable elements, including taper, apex and homogeneity. Despite technological advances in instrumentation, assessing the quality of these procedures remains challenging due to anatomical variations and technique sensitivity [6]. AI models tailored for dentistry, especially those capable of interpreting root canal images, have shown promise in providing more objective and less biased assessments of the root canal anatomy [7] . Narrative reviews of AI chatbots in endodontic education emphasize their potential to enhance personalized learning and clinical reasoning while also noting concerns about hallucinations, biases, and ethical implications[8]. These reviews advocate the development of validated case-specific chatbot solutions aligned with expert judgments. Similarly, a scoping review of dental education literature highlighted the roles of AI, virtual reality (VR), and augmented reality (AR) in improving student engagement, despite ongoing methodological and implementation challenges. A more focused review of endodontic education further demonstrated AI's utility of AI in radiographic interpretation, treatment planning, preclinical simulation, and performance evaluation, identifying multiple emerging educational applications [8].

ChatGPT-4o and MedGebra GPT-4o have demonstrated notable accuracy and consistency by correctly answering up to 90% of multiple-choice questions in endodontics, while GPT-3.5 also exhibited high validity and reliability (Cronbach’s alpha > 0.7) in responding to frequently asked questions, outperforming other chatbots such as Google Bard and Bing; these findings underscore the promising potential of artificial intelligence applications in the field of endodontics [9]. In a 2024 study, ChatGPT-4 achieved 99% accuracy in diagnosing pulpal and apical pathoses—surpassing both junior and senior dental students, who averaged approximately 78%—though the authors cautioned that over-reliance on AI may impede the development of clinical reasoning skills [8].

Despite the potential of LLMs for diagnosing psychomotor skills, there is a lack of exploration in this area. This pilot study investigates ChatGPT-4 as a reliable aid for expert evaluators in assessing undergraduate endodontic performance. A structured rubric focuses on taper, apical preparation, homogeneity and iatrogenic error prevention. Intraclass Correlation Coefficients (ICC) are used to evaluate agreement levels. ICC are widely used and validated measures of inter-rater reliability in healthcare education.

This pilot exploratory study aimed to determine the concordance between ChatGPT-4 and an expert endodontist in rating undergraduate root-canal shaping using a predefined five-item rubric (canal centering, homogeneity, procedural errors, apical shaping, and overall taper). The primary outcome was agreement between raters—quantified with intraclass correlation coefficients and weighted kappa—to appraise the feasibility of incorporating large language models as a supportive tool in competency-based, preclinical dental education.
Because prior work in dental education has focused mainly on diagnostic tasks, the role of generative models in evaluating psychomotor performance remains underexplored; this study addresses that gap by testing feasibility rather than providing definitive validation[3] . We hypothesize that AI-generated assessments do not significantly differ from expert evaluations in dental education, suggesting its potential for reliable integration into competency-based training.

**2. Methods**

**2.1 Study Design and Setting**

This cross-sectional pilot study was conducted at the Department of Endodontics, Faculty of Dentistry, Istanbul Aydın University, during the 2024–2025 academic years. The study was embedded in a clinical training program for fourth- and fifth-year dental students with the aim of evaluating the feasibility of integrating AI-assisted assessment into endodontic education.

**2.2 Participants and Case Selection**

A convenience sample of fourth- and fifth-year dental students who underwent nonsurgical root canal treatment (RCTs) as part of their routine clinical duties was invited to participate voluntarily. The inclusion criteria required completed root canal treatments with available preoperative and postoperative periapical radiographs, procedures performed entirely by a single student under supervision, and radiographs of sufficient quality for evaluation. Patients with missing or unreadable radiographs, severe anatomical anomalies, or incomplete canal fillings were excluded. A total of 32 patients, each contributing one final periapical radiograph, were included in the analysis.


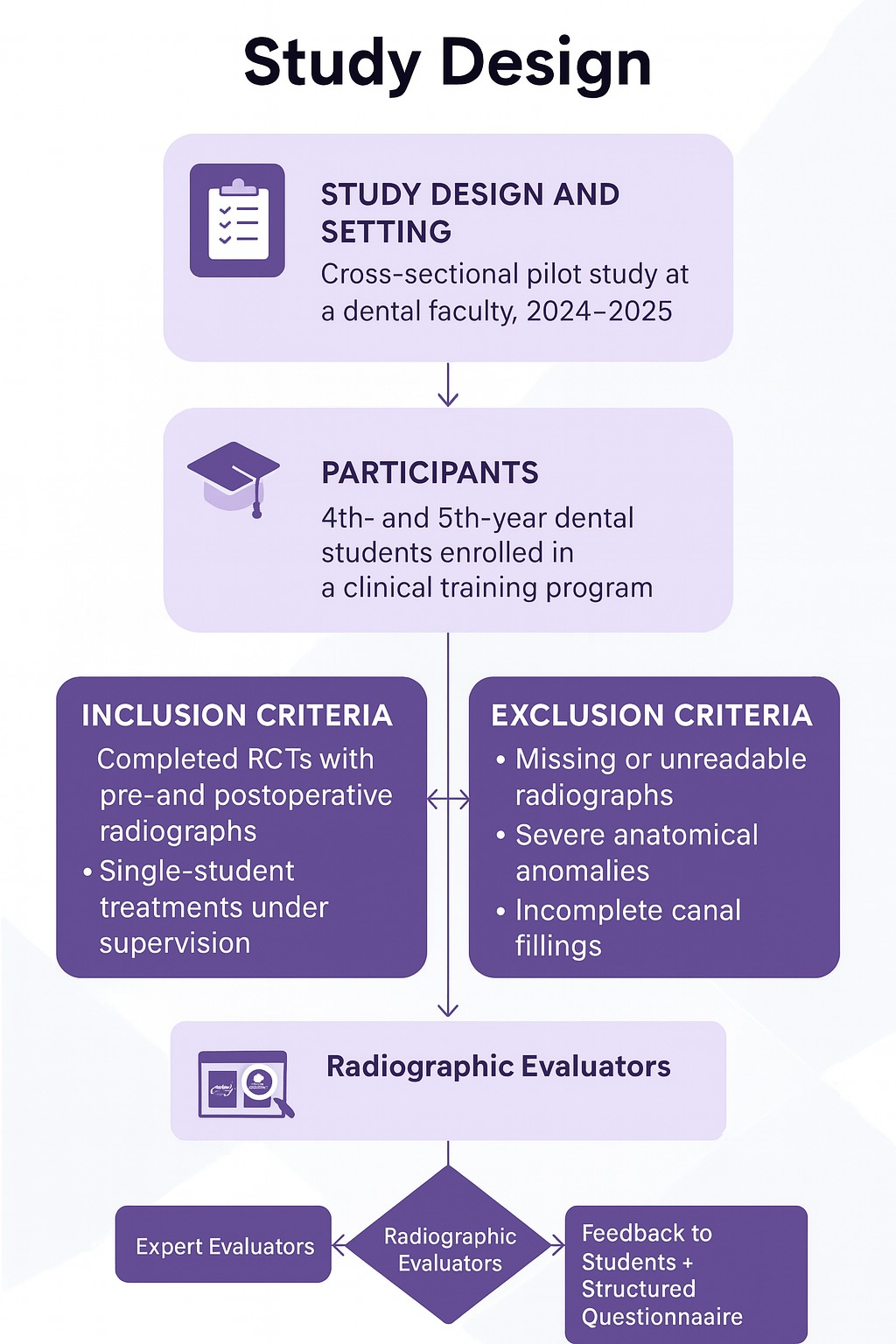


**Figure 1.** Study design and evaluation workflow for AI-assisted radiographic assessment in endodontic education.

**2.3 Radiographic Assessment and Scoring**

Preoperative and postoperative periapical radiographs were anonymized and coded for each patient. The radiographs were independently evaluated by one endodontist with ten years of professional experience and a GPT-4o based artificial intelligence model using a standardized scoring rubric. Both the expert evaluator and the AI model applied the same predefined rubric, ensuring consistency and comparability across all assessments.

The following five evaluation parameters were assessed:

1. Canal centering – alignment and deviation relative to the original canal path
2. Homogeneity – radiographic presence of canal opacity or uncleaned areas
3. Overextension/perforation – signs of extrusion or iatrogenic damage
4. Apical shaping – taper and finish at the apical third
5. Overall shaping and taper – continuity and quality throughout the canal length

Each criterion was rated on a 5-point Likert scale (1 = very poor to 5 = excellent). The expert evaluators were blinded to student identities and scores. The AI model was programmed to interpret radiographic inputs using a structured prompt set, and to generate both score outputs and narrative feedback.

The postoperative root canal treatment radiographs were downloaded as JPG image files and subsequently evaluated by ChatGPT-4o using a structured prompt-based assessment. The model was guided by the following prompts.

Postoperative root canal treatment radiographs were evaluated by ChatGPT-4o using structured prompts to assess five specific criteria—canal centering, homogeneity, procedural errors, apical shaping, and overall taper—on a 5-point Likert scale (1 = very poor, 5 = excellent), with a brief justification provided for each score and an overall average calculated.

All AI model evaluations were conducted using a standardized prompt: *“Please provide a brief justification for each score and calculate the overall average.”* No follow-up interaction, clarification, or prompt refinement was permitted beyond this initial instruction. All responses were recorded in their original form and stored for subsequent analysis.

**2.4 Student Feedback Collection**

After completing the clinical procedures and radiographic evaluations, the participating students received feedback based on the expert and AI evaluations. The students were then asked to complete a structured questionnaire that compared the two feedback formats in terms of clarity, educational value, practicality, and overall preferences. The questionnaire used a 5-point Likert scale and included open-ended questions for qualitative analysis. The questionnaire was specifically developed for this study, and an English version has been provided as Supplementary File 1.outcom

**2.5 Statistical Analysis**

All statistical analyses were conducted to compare expert- and AI-based assesment outcomes across three domains: student perceptions, scoring correlation, and inter-rater reliability.

To assess differences in student perceptions of expert versus AI-generated feedback, participants (n = 32) completed a structured questionnaire evaluating three categories: educational contribution, clinical thinking support, and perceived reliability. Responses were recorded using a 5-point Likert scale (1 = strongly disagree, 5 = strongly agree). Since each participant rated both feedback sources, paired-sample t-tests were conducted to compare mean scores between expert and AI conditions. Statistical significance was set at p < 0.05.

To examine the linear relationship between expert and AI performance scores, Pearson correlation coefficients (r) were calculated across five rubric-based assessment criteria: canal centering, homogeneity, procedural errors, apical shaping, and overall taper. Correlations were interpreted as weak when r < 0.3, and associations were considered statistically non-significant if p > 0.05.

To evaluate inter-rater reliability further, Intraclass Correlation Coefficients (ICC) were computed for the same five performance criteria. ICC values were interpreted based on established thresholds: < 0.5 = poor agreement, 0.5–0.75 = moderate agreement; and > 0.75 = good to excellent agreement. These analyses quantified the consistency between AI-generated and expert scores across core endodontic performance dimensions.

For categorical respons to the multiple-choice item “Which system was more beneficial for learning?” (n=32), a Chi-square goodness-of-fit test was conducted to determine whether the distribution of preferences differed significantly from uniform expectation. Statistical significance was set at p < 0.05. All analyses were conducted using SPSS version 27.0, and two-tailed p-values < 0.05 were considered statistically significant.

A priori power analysis was performed using G*Power 3.1. Based on the observed intraclass correlation coefficients (ICC = 0.36–0.45) and Pearson correlation coefficients (<0.30), the effect size was calculated as 0.60. With an alpha error probability of 0.05 and a power value (1-β) of 0.80, the required total sample size was estimated as 30. Accordingly, our study included 32 students, thereby meeting the minimum sample size requirement for adequate statistical power.

**3. RESULT**

**3.1 Inter-Rater Reliability Assessment**

Intraclass correlation coefficients (ICC) were used to assess the degree of agreement between expert and AI-based evaluations. ICC values ranged from 0.36 to 0.45, indicating poor to moderate agreement (Figure 2). The highest ICC was observed for procedural errors (ICC = 0.45), suggesting relatively better alignment between raters in this domain. The lowest agreement was found for the evaluation of the overall taper (ICC = 0.36). These findings suggest a limited inter-rater reliability. Further development is required to improve the consistency of AI-based scoring with expert judgment in detailed endodontic assessments.

# **Table 1**. Intraclass Correlation Coefficients (ICC) Between Expert and AI Scores

| Performance Criteria | ICC Value |
| --- | --- |
| Canal Centering | 0.42 |
| Homogeneity | 0.38 |
| Procedural Errors | 0.45 |
| Apical Shaping | 0.40 |
| Overall Taper | 0.36 |

Note: ICC interpretation thresholds – values <0.5 indicate poor agreement, 0.5–0.75 indicate moderate agreement, and >0.75 indicate good to excellent agreement.

In addition, Cohen’s kappa coefficients indicated consistently low agreement across all criteria (Table 2).

## **Table 2**. Cohen's Kappa for Agreement Between AI and Expert

| **Criterion** | **Cohen's Kappa** |
| --- | --- |
| Canal Centering | 0.038 |
| Homogeneity | -0.156 |
| Overextension/Perforation | 0.055 |
| Apical Shaping | 0.084 |
| Overall Taper | 0.121 |

Cohen’s kappa coefficients for each performance criterion. All values indicated low agreement (κ < 0.4), supporting the limited consistency observed between AI and expert ratings.

**
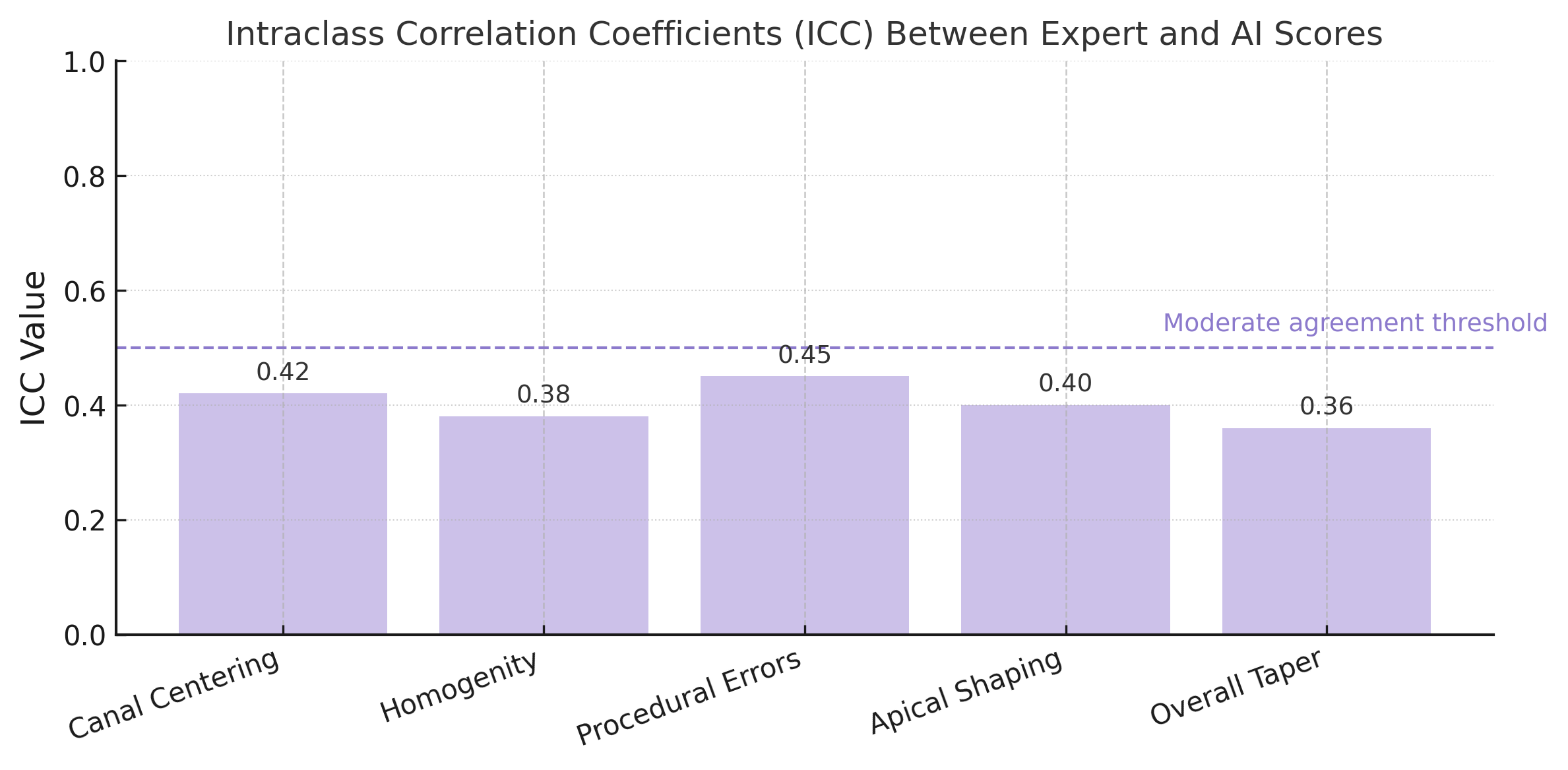
**

**Figure 2.** Intraclass Correlation Coefficients (ICC) Between Expert and AI Scores

*(ICC values were interpreted based on commonly accepted thresholds: values below 0.5 indicate poor agreement, 0.5–0.75 moderate agreement, 0.75–0.9 good agreement, and above 0.9 excellent agreement.)*

**3.2 AI–Expert Score Correlation**

Pearson correlation coefficients were calculated to examine the relationship between expert and AI scoring across performance criteria. All correlations were weak (r < 0.3) and statistically non-significant (p > 0.05), indicating that AI scoring did not consistently align with expert scoring in terms of linear trends, particularly in categories such as canal centering, homogeneity, and apical shaping (Figure 3).

**Table 2. Pearson Correlation Coefficients and p-values Between Expert and AI Scores**

| Performance Criteria | Pearson r | p-value |
| --- | --- | --- |
| Canal Centering | 0.21 | 0.14 |
| Homogeneity | 0.19 | 0.18 |
| Procedural Errors | 0.24 | 0.12 |
| Apical Shaping | 0.26 | 0.10 |
| Overall Taper | 0.22 | 0.17 |

Note: Correlation coefficients (r) < 0.3 were interpreted as weak, and p-values > 0.05 were considered not statistically significant.


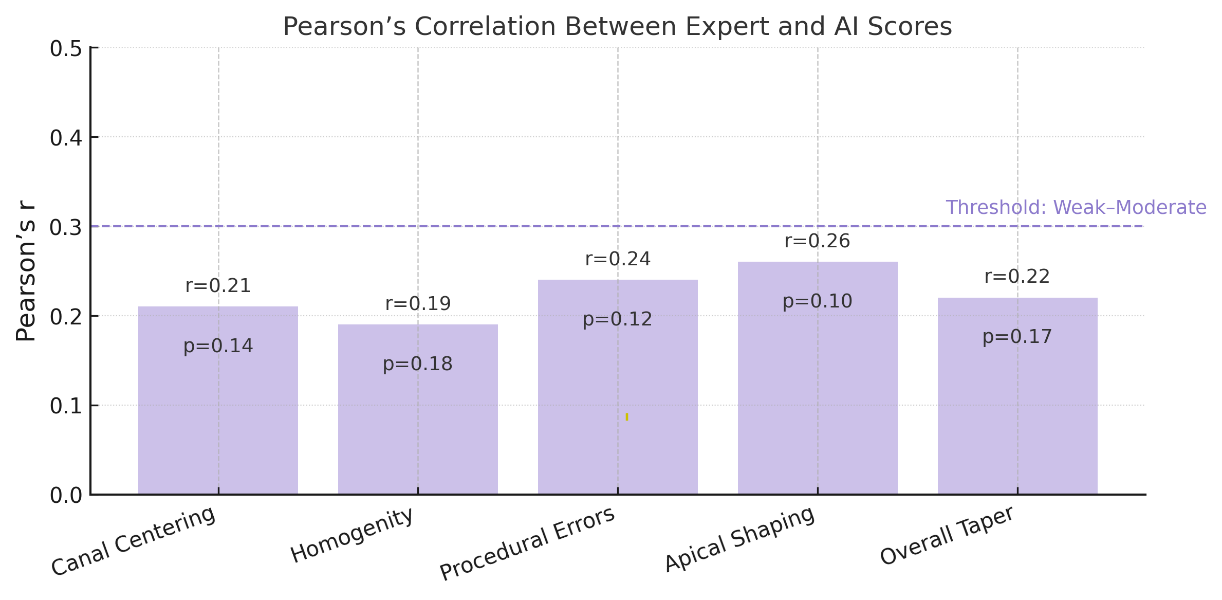


**Figure 3.** Correlation Between Expert and AI Scores Across Performance Criteria

To provide more robust estimates, 1,000 bootstrap resamples were performed. The 95% CIs confirmed weak and non-significant correlations across all criteria (Table 3)

## **Table 3.** Bootstrap Estimates of Pearson Correlation (95% CI)

| **Criterion** | **Mean Pearson r** | **95% CI Lower** | **95% CI Upper** |
| --- | --- | --- | --- |
| Canal Centering | 0.02 | -0.341 | 0.371 |
| Homogeneity | -0.039 | -0.396 | 0.317 |
| Overextension/Perforation | -0.04 | -0.37 | 0.312 |
| Apical Shaping | 0.124 | -0.251 | 0.479 |
| Overall Taper | 0.164 | -0.169 | 0.457 |

Bootstrap resampling (1,000 iterations) was used to estimate 95% confidence intervals for Pearson correlations between AI and expert scores. The results confirmed weak and non-significant associations across all performance criteria.

**3.3 Bland–Altman Analysis**

Bland–Altman analysis further highlighted systematic bias and wide limits of agreement, suggesting poor interchangeability between AI and expert scoring. On average, AI tended to assign slightly higher scores than the expert evaluator across most criteria, although this difference was inconsistent across cases. (Table 4).

## **Table 4**. Bland–Altman Analysis for Agreement Between AI and Expert Scores

| **Criterion** | **Mean Difference** | **Lower LoA (95%)** | **Upper LoA (95%)** |
| --- | --- | --- | --- |
| Canal Centering | 1.0 | -0.79 | 2.79 |
| Homogeneity | 0.21 | -1.99 | 2.4 |
| Overextension/Perforation | 1.58 | -1.6 | 4.76 |
| Apical Shaping | 0.52 | -1.65 | 2.68 |
| Overall Taper | 0.63 | -0.87 | 2.13 |

Bland–Altman analysis showing mean differences (bias) and limits of agreement (LoA) between expert and AI scores. The wide LoA indicate poor interchangeability between the two evaluators. On average, AI tended to assign slightly higher scores than the expert evaluator across most criteria, although agreement remained poor.

**3.4 Student Ratings of Feedback Sources**

A total of 32 students evaluated both expert and AI-generated feedback across three domains: educational contribution, clinical thinking support, and perceived reliability. Expert feedback consistently received higher mean scores than AI feedback in all categories. Educational contribution scored 4.29 (expert) versus 3.90 (AI), clinical thinking support 4.19 versus 4.06, and perceived reliability 4.00 versus 3.91. Although expert feedback was rated more favorably, no statistically significant differences were observed (p > 0.05), indicating that students found AI feedback moderately acceptable (Figure 4).

# **Table 4**. Comparison of Student Ratings: Expert vs. AI Feedback

|  | Expert (Mean Rating) | AI (Mean Rating) | p-value |
| --- | --- | --- | --- |
| Educational Contribution | 4.29 | 3.90 | 0.072 |
| Clinical Thinking Support | 4.19 | 4.06 | 0.475 |
| Perceived Reliability | 4.00 | 3.91 | 0.614 |

Note: Mean ratings were compared using paired-sample t-tests. Differences were not statistically significant (all p-values > 0.05).

**
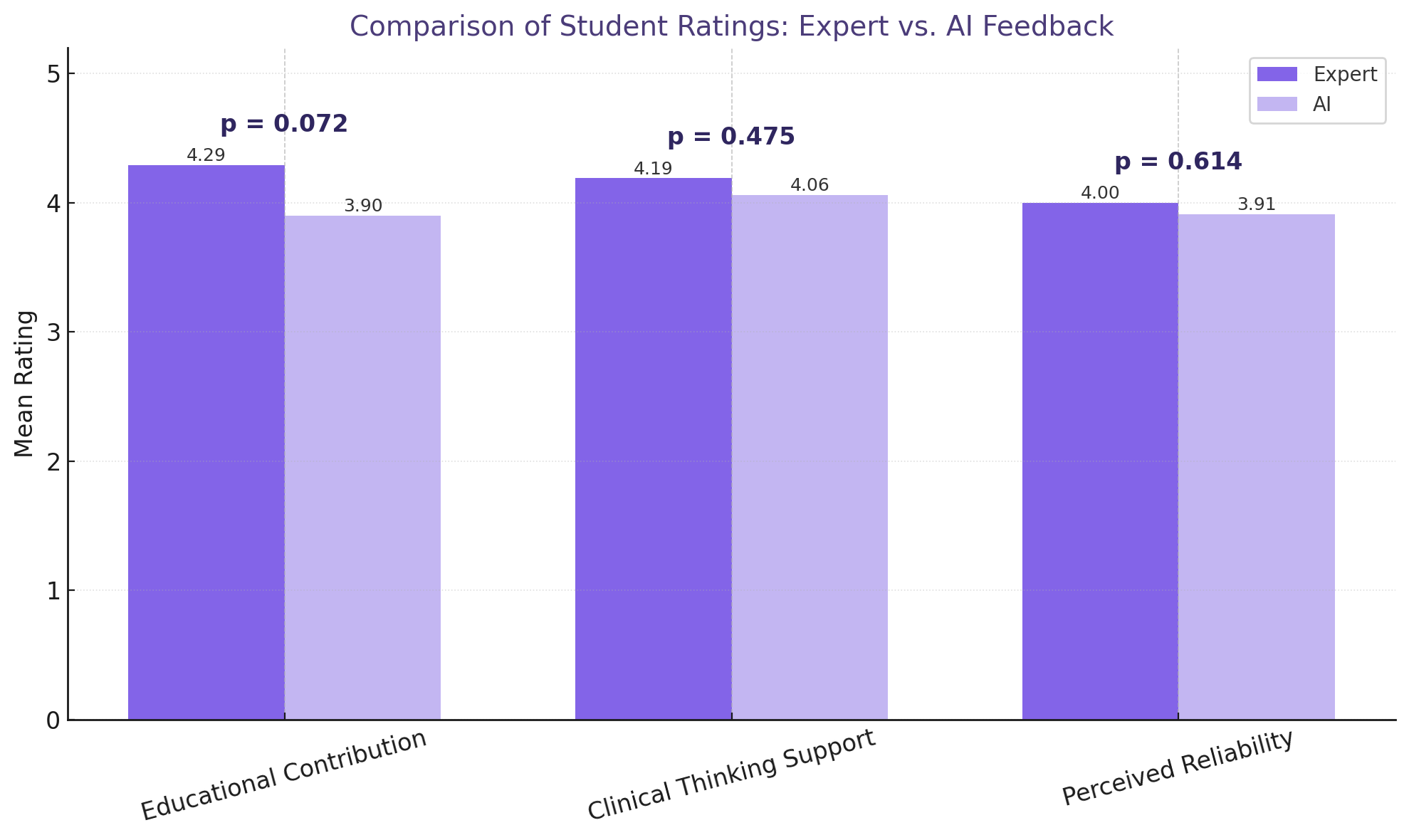
**

**Figure 4.** Comparison of Student Ratings for Expert vs. AI Feedback (n = 32)

**3.5 Student Suggestions for Improving Feedback Systems**

Of the 32 participating students, 25 responded to the open-ended feedback questions. When these responses were grouped through content analysis, 44% of the students offered unique and heterogeneous suggestions. The remaining responses clustered around key improvement areas, including the need to incorporate clinical context or patient history, clearer explanation logic in AI-generated feedback, and the integration of interactive or comparative features (Figure 5).

**
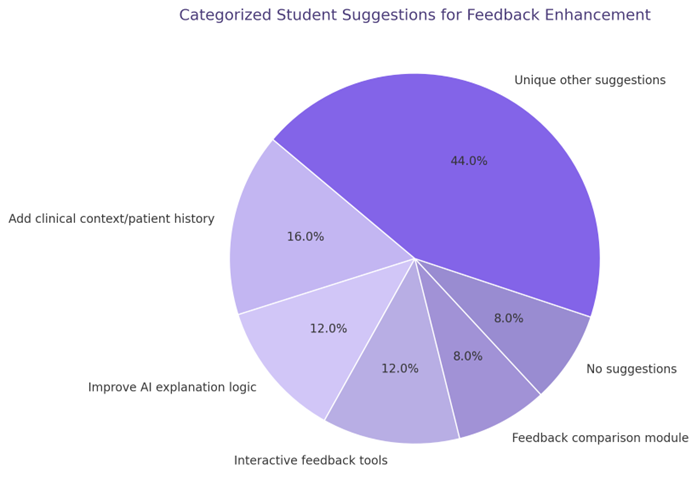
**

**Figure 5.** Student Suggestions for Feedback Enhancement

*(Figure 5. Categorized suggestions provided by students in response to open-ended feedback questions. Out of 32 participants, 25 provided responses. Among them, 2 students stated they had no suggestions. The remaining responses were grouped into thematic categories, including a set of 11 unique individual suggestions that did not overlap with others.)*

**3.6 Perceived Educational Benefit of Feedback Systems**

Participants’ responses to the question “*Which system was more beneficial for learning?*” were categorized based on open-ended feedback from 32 students. Most respondents (53.1%) favored the combined use of expert and AI feedback, whereas smaller proportions preferred either the expert (9.4%) or AI (9.4%) system alone. The remaining responses (28.1%) were mixed or did not specify a clear preference. Percentages were calculated based on all 32 responses (Figure 6).

**
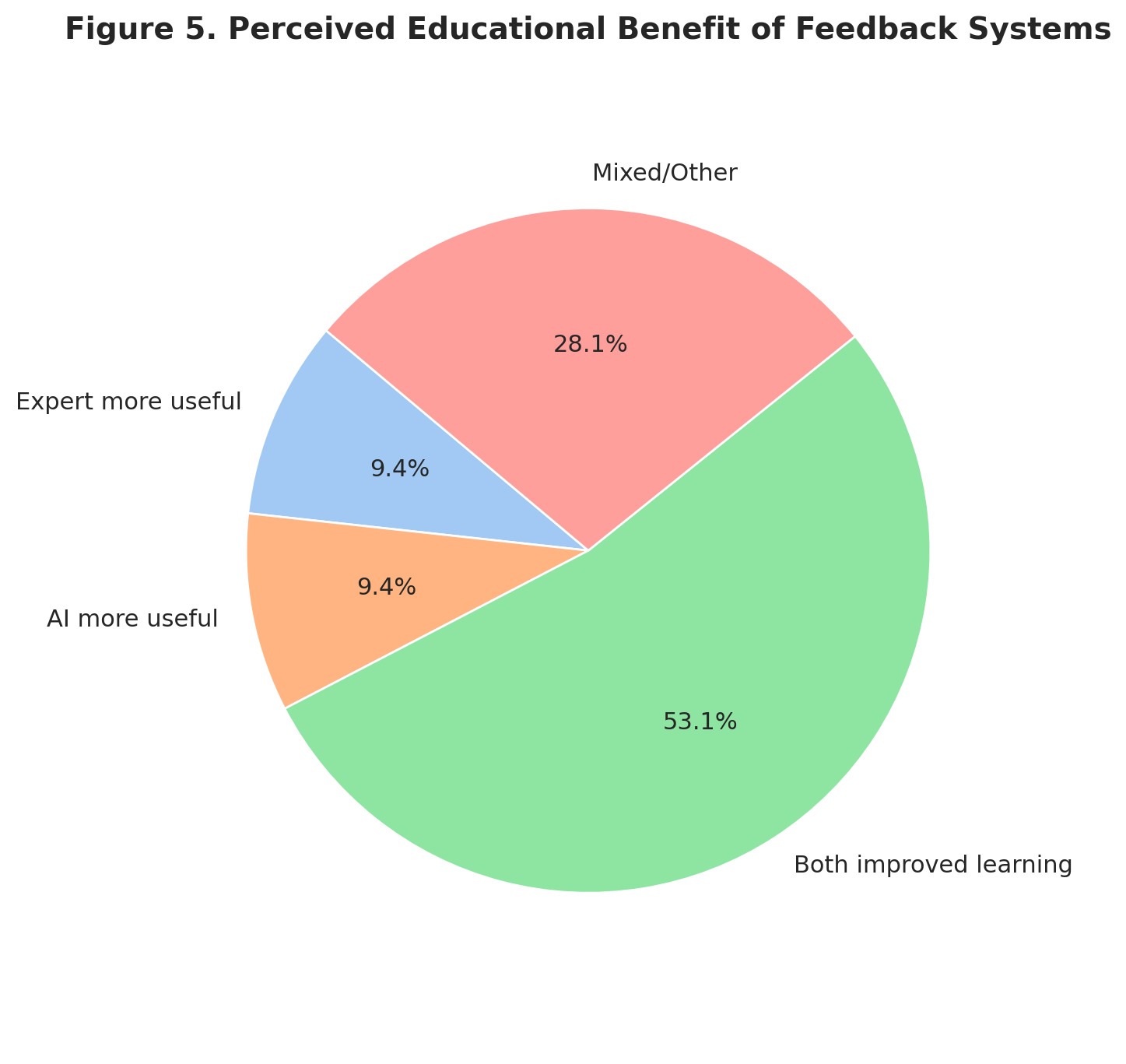
**

**Figure 6.** Perceived Educational Benefit of Feedback Systems

**3.7 Illustrative Student Comments**

In addition to the categorized responses, several open-ended comments offered further insight into students’ perceptions of the feedback systems. These statements reflect the perceived strengths and limitations of both modalities:

*“AI feedback was fast but lacked depth in explanation.”*
*“Expert feedback helped me understand clinical reasoning better.”*
*“Using both gave a balanced perspective and improved my learning.”*

**4. Discussion**

This study evaluated the agreement between AI-based scoring using ChatGPT-4o and expert assessments of undergraduate endodontic performance. While students found AI feedback moderately useful, our quantitative analysis showed limited alignment between AI and expert evaluations. Specifically, intraclass correlation coefficients (ICC) ranged from 0.36 to 0.45, indicating poor to moderate inter-rater reliability. Pearson correlation coefficients for all five performance criteria were below 0.3 and non-significant, suggesting no consistent linear relationship between AI and expert scores.

The low ICC values highlight a lack of scoring consistency between AI and human evaluators, particularly in tasks involving fine procedural distinctions. The highest agreement was observed in the category of procedural errors (ICC = 0.45), likely due to the detectability of gross mistakes. However, lower agreement in taper assessment (ICC = 0.36) underscores the AI’s limitations in evaluating geometrically subtle or context-dependent performance elements.

These results align with prior studies indicating that while AI systems may offer standardized outputs, they often fail to replicate the nuanced judgment of human experts. For instance, in simulation-based training, AI evaluation of clinical skills showed weak to moderate alignment with expert ratings, especially in complex tasks involving dexterity and adaptability [11] . This gap is largely attributed to differences in evaluative focus: AI systems tend to rely on measurable and procedural features (e.g., pixel-level analysis), whereas human experts incorporate clinical reasoning, adaptability, and contextual interpretation into their assessments. Previous studies reinforce the context-dependent reliability of AI in dental education. Ali et al. [12] reported that ChatGPT can provide satisfactory accuracy in knowledge-based assessments, while also cautioning against overreliance due to risks of misinformation[12]. Hatia et al. [13] found that ChatGPT achieved high accuracy and completeness when responding to orthodontic clinical scenarios, yet emphasized that it should be regarded as a supportive rather than substitutive tool for expert reasoning[13]. Demir et al. [14] highlighted that AI performance evolves over time (e.g., GPT-3.5 vs. GPT-4) and that reliability is influenced by inter-rater consistency and prompt engineering, underscoring the methodological nuances that determine AI’s educational value. Together, these findings align with our results, suggesting that while LLMs may enrich dental training, their role is bounded by task complexity, model evolution, and the critical need for expert oversight[14].

From a psychometric standpoint, the absence of strong correlation metrics suggests that ChatGPT-based evaluations, in their current form, should not replace expert judgment. Instead, they may serve as supplementary tools, providing preliminary insights that can be verified or contextualized by experienced clinicians. As previously proposed in AI-based educational models, improving alignment would require calibration with expert-annotated datasets, incorporating case variability, and adopting human-in-the-loop validation frameworks [15].

Although our findings indicate low correlation between AI and expert assessments, several underlying factors may explain this discrepancy. First, ChatGPT-4 is a text-based large language model (LLM) and cannot interpret radiographic data directly, which is central to endodontic evaluation (e.g., taper formation, voids, apical shaping). Without image processing, subtle but clinically important details may be overlooked. Second, expert evaluators incorporate broader clinical context and tacit knowledge—including anatomical variability, patient history, and intra-operative cues—that AI models cannot replicate. Third, AI performance is also influenced by factors such as prompt design, domain-specific training data, and model version, which may explain variability in reliability across tasks [16]Finally, although differences in student perceptions of expert versus AI feedback were not statistically significant, even small discrepancies may have pedagogical importance. Prior research demonstrates that student trust, motivation, and the perceived credibility of feedback strongly influence how learners engage with and act on feedback, ultimately shaping learning behaviors and performance outcomes [17].

Furthermore, the limitations of text-only language models such as ChatGPT-4 become particularly evident in endodontics, where visual interpretation of radiographic data is central to clinical judgment. Recent studies have explored multimodal AI systems such as GPT-4V and Gemini Pro in dentistry, particularly for radiographic tasks. Aşar et al. [18]showed that a customized GPT-4V outperformed general models in detecting supernumerary teeth on periapical radiographs, highlighting the potential of domain-specific fine-tuning [18]. Similarly, Fukuda et al. [19] compared GPT-4V and Gemini Pro in the Japanese National Dental Examination and found that, although GPT-4V achieved higher accuracy overall, performance declined with increasing image complexity, underlining the limitations of current multimodal systems in handling complex visual data [19]. In addition, Brin et al. [20] reported that while GPT-4V reliably recognized imaging modalities, its diagnostic accuracy in pathology detection was inconsistent, suggesting that multimodal AI still requires rigorous validation before widespread adoption in dental education and assessment [20] . Although our study focused on text-based outputs, many key aspects of root canal evaluation—such as taper formation, transportation, or void detection—rely on image analysis. This limitation has also been noted in other educational contexts [21] reported that 45% of items in the Japanese National Dental Examination had to be excluded from AI evaluation due to image content, underscoring the inherent constraints of current LLMs in visually dependent domains.

In this regard, emerging multimodal AI models—capable of processing text, images, and radiographs—represent a promising direction, though their integration into educational assessment remains in early stages. Studies exploring these systems in dental radiology have shown potential but also inconsistent performance in complex diagnostic scenarios [22, 23]. Until such models are adequately validated, AI-generated assessments should be interpreted with caution, especially in radiographically intensive specialties such as endodontics.

Despite the observed inconsistencies between AI and expert evaluations, students in our study rated both sources of feedback as valuable. Expert feedback received marginally higher average scores across all domains—educational contribution (4.29 vs. 3.90), clinical thinking (4.19 vs. 4.06), and perceived reliability (4.00 vs. 3.91)—although none of these differences reached statistical significance. These findings suggest a subtle learner preference for expert evaluations, possibly reflecting trust in human authority rather than objective performance differences. Although AI and expert evaluations showed limited agreement, students still perceived AI-generated feedback as useful. This apparent contradiction can be explained by the distinct advantages offered by each feedback source.

This preference is consistent with what has been termed the “status effect,” where learners inherently assign greater credibility to human evaluators, especially in formative settings. Nonetheless, AI-generated feedback was not dismissed. Students acknowledged its utility, particularly for its speed, objectivity, and consistency. This aligns with previous research reporting that while students rated human feedback higher in clarity and reliability, a majority still saw AI as a valuable complementary tool[24, 25].

Importantly, 53.1% of our participants indicated a preference for receiving both AI and expert feedback, supporting a hybrid educational model. This dual-feedback approach leverages the scalability and standardization of AI with the contextual richness and adaptability of expert input. From a pedagogical standpoint, such hybrid systems may help alleviate faculty workload while maintaining educational quality, particularly in large cohorts or resource-limited environments [26].

However, students’ open-ended feedback also highlighted key limitations. Several respondents questioned the depth and interpretability of AI-generated comments. This points to a broader issue in AI-assisted education: the need for explainable and interactive feedback. Previous studies [24, 27] have highlighted the limitations of black-box AI in clinical education, noting that non-transparent outputs can obscure underlying reasoning and impede learning. The integration of explainable AI frameworks, such as LIME or SHAP, may enhance learner trust and understanding in future applications [24].

Local Interpretable Model-Agnostic Explanations (LIME), introduced by Ribeiro et al. in 2016, is a widely used model-agnostic explainable AI (XAI) algorithm. Its primary strength lies in providing interpretable, local explanations for individual predictions across a broad range of machine learning models, including complex architectures like deep neural networks and ensemble methods. By approximating the original model’s decision boundary with a simpler, interpretable surrogate model in the vicinity of a single instance, LIME enables users to understand the factors influencing specific predictions. For example, in clinical contexts, LIME can highlight which patient features, such as certain symptoms, contributed to a particular diagnosis suggested by the model. This versatility and transparency make LIME especially valuable for supporting decision-making in high-stakes environments like healthcare [28].

SHapley Additive exPlanations (SHAP), introduced by Lundberg et al., represents a major advancement in model interpretability by providing a unified, model-agnostic framework for explaining individual predictions. Unlike earlier XAI approaches that were often tailored to specific model types, SHAP assigns importance values to each feature for a given prediction, effectively highlighting the factors that most influence the model’s output. Its flexibility allows SHAP to be applied across a range of machine learning algorithms, including both deep learning and tree-based models. Notably, SHAP excels in complex scenarios with many interacting features, often outperforming alternative methods in uncovering nuanced relationships within the data. By enabling a clearer understanding of how and why specific predictions are made, SHAP has become a valuable tool for advancing transparency and trust in machine learning applications[29] .

In this study, student perceptions reinforce the idea that AI-based assessment tools should be designed to augment, rather than replace, expert feedback. They can offer a scalable and supportive mechanism that can contribute to skill development, particularly in foundational and intermediate stages of clinical training.

Given the limited agreement between AI and expert assessments observed in this study, future research should focus on refining AI models to better reflect expert judgment in procedural domains. One promising approach involves the development of calibration datasets drawn from expert-scored clinical cases, which can help train models to account for variability in technique and interpretation.

Another critical step is the incorporation of multimodal AI systems capable of processing both textual and visual data. Since many key endodontic competencies rely heavily on radiographic interpretation, the ability to integrate image-based features into AI assessments is essential for broader applicability. Pilot efforts using such models in dental education are ongoing, but further validation is needed before widespread implementation [9].

Human-in-the-loop frameworks, where AI outputs are reviewed or modified by educators, may also improve reliability and educational value. These models could strike a balance between automation and clinical expertise, particularly in formative assessments. Additionally, incorporating explainability mechanisms into AI-generated feedback may enhance learner trust and promote deeper engagement with the evaluation process.

Larger-scale studies involving diverse student cohorts, multiple institutions, and different clinical tasks will be valuable for generalizing findings and informing best practices for AI integration in dental education. Despite these strengths, certain limitations should be acknowledged. This study was conducted at a single institution with a relatively small sample size (n = 32), and although a priori power analysis was performed, the achieved statistical power remained modest. The use of a single expert as the reference standard, while ensuring consistency, introduces potential subjective bias. Moreover, the applied model was a general-purpose, text-based language model without radiographic processing capabilities, which restricted its ability to capture image-dependent endodontic parameters. Finally, the outputs of large language models are inherently influenced by prompt design and training data, which may introduce variability in scoring. Taken together, these constraints reinforce the exploratory nature of our findings and highlight the need for larger, multicenter studies with diverse student cohorts and multiple expert evaluators. Future research should also prioritize the development of domain-specific, multimodal AI models capable of integrating both textual and radiographic data, alongside human-in-the-loop frameworks to improve reliability, transparency, and educational value in endodontic education. Another important consideration is the rapid evolution of large language models. ChatGPT has already advanced to version 5, which is currently being widely adopted by researchers across various fields. Outcomes of studies employing ChatGPT may therefore differ depending on the specific version utilized. To enhance the generalizability, citation potential, and academic impact of future research, it is essential to explicitly acknowledge version-related variability and to complement current findings with updated or comparative analyses involving newer model iterations.

**5. Conclusion**

This pilot exploratory study examined the concordance between ChatGPT-4o and expert evaluations. While the sample size was modest, the design was intended as a proof-of-concept, aiming to assess feasibility rather than to provide definitive validation. While students perceived AI-generated feedback as moderately useful, the quantitative findings revealed only poor to moderate agreement, with limited correlation between AI and expert scores. These results suggest that, in its current form, AI cannot substitute for expert judgment but may serve as a supplementary tool to support formative assessment in dental education. The findings highlight both the potential and the current limitations of text-based large language models, particularly their inability to process radiographic data and incorporate clinical context. Future research should focus on the development of multimodal, domain-specific models, larger multicenter cohorts, and human-in-the-loop frameworks to enhance reliability, transparency, and educational value. Our findings should be interpreted within the context of a pilot exploratory design. This study primarily serves as a proof-of-concept to evaluate feasibility, and not as a conclusive validation of AI applicability in endodontic education.

**Abbreviations:**

AI: Artificial intelligence

ICC: Intraclass correlation coefficients

LLM: Large language models

VR: Virtual reality

AR: Augmented reality

RCT: Nonsurgical root canal treatment

XAI: Explainable AI

SHAP: SHapley Additive exPlanations

LIME: Local Interpretable Model-Agnostic Explanations

**Declarations**

**Ethics approval and consent to participate**

This study was approved by the Ethics Committee of Istanbul Aydın University (approval number: 144/2025). All participating students provided informed consent, and patient-related data were anonymized in accordance with ethical research principles and institutional regulations.

All participants provided written informed consent prior to participation in the study.

This study was conducted in accordance with the principles of the Declaration of Helsinki.

**Consent for publication**

Not applicable.

**Availability of data and materials**

The datasets used and analyzed during the current study will be made available from the corresponding author on reasonable request.

**Competing interests**

The authors declare that they have no competing interests.

**Funding**

No funding was provided for this study.

**Authors' contributions**

All authors have significantly contributed to the work both directly and intellectually and have given their approval for publication.

**Declaration of generative AI and AI-assisted technologies in the writing process**

During the preparation of this manuscript, the authors used ChatGPT-4 to improve the clarity and readability of the text. All generated suggestions were carefully reviewed and revised by the authors, who take full responsibility for the final content.

**Trial registration**

Not applicable. This study is observational and not a clinical trial.

**References**

1. Karobari MI, Adil AH, Basheer SN, Murugesan S, Savadamoorthi KS, Mustafa M, et al. Evaluation of the Diagnostic and Prognostic Accuracy of Artificial Intelligence in Endodontic Dentistry: A Comprehensive Review of Literature. Computational and Mathematical Methods in Medicine. 2023;2023. https://doi.org/10.1155/2023/7049360.

2. Isaza-Restrepo A, Gómez MT, Cifuentes G, Argüello A. The virtual patient as a learning tool: A mixed quantitative qualitative study. BMC Med Educ. 2018;18. https://doi.org/10.1186/s12909-018-1395-8.

3. Qutieshat A, Al Rusheidi A, Al Ghammari S, Alarabi A, Salem A, Zelihic M. Comparative analysis of diagnostic accuracy in endodontic assessments: dental students vs. artificial intelligence. Diagnosis (Berl). 2024;11:259–65. https://doi.org/10.1515/dx-2024-0034.

4. Kung TH, Aggabao R, Maningo J, Tseng V, Elepaño C, Sillos C, et al. Performance of ChatGPT on USMLE: Potential for AI-assisted medical education using large language models. PLOS digital health. 2023;2:e0000198. https://doi.org/10.1371/journal.pdig.0000198.

5. Krishnappa P, Sriranga J, Shwetha K, Sagarkar A, Singh A. Faculty Experience and Reliability of Assessing Narrative Reports using Rubrics: Report from a Dental School in India. Journal of Indian Association of Public Health Dentistry. 2023;21:76–81. https://doi.org/10.4103/jiaphd.jiaphd_76_22.

6. Versiani MA, Martins JNR, Ordinola-Zapata R. Anatomical complexities affecting root canal preparation: a narrative review. Australian Dental Journal. 2023;68:S5–23. https://doi.org/10.1111/adj.12992.

7. Parinitha MS, Doddawad VG, Kalgeri SH, Gowda SS, Patil S. Impact of Artificial Intelligence in Endodontics: Precision, Predictions, and Prospects. J Med Signals Sens. 2024;14:25. https://doi.org/10.4103/jmss.jmss_7_24.

8. Mohammad-Rahimi H, Sohrabniya F, Ourang SA, Dianat O, Aminoshariae A, Nagendrababu V, et al. Artificial intelligence in endodontics: Data preparation, clinical applications, ethical considerations, limitations, and future directions. Int Endod J. 2024;57:1566–95. https://doi.org/10.1111/iej.14128.

9. Mohammad-Rahimi H, Ourang SA, Pourhoseingholi MA, Dianat O, Dummer PMH, Nosrat A. Validity and reliability of artificial intelligence chatbots as public sources of information on endodontics. Int Endod J. 2024;57:305–14. https://doi.org/10.1111/iej.14014.

10. Claman D, Sezgin E. Artificial Intelligence in Dental Education: Opportunities and Challenges of Large Language Models and Multimodal Foundation Models. JMIR Med Educ. 2024;10:e52346. https://doi.org/10.2196/52346.

11. Cold KM, Agbontaen K, Nielsen AO, Andersen CS, Singh S, Konge L. Artificial intelligence for automatic and objective assessment of competencies in flexible bronchoscopy. J Thorac Dis. 2024;16:5718–26. https://doi.org/10.21037/jtd-24-841.

12. Ali K, Barhom N, Tamimi F, Duggal M. ChatGPT—A double-edged sword for healthcare education? Implications for assessments of dental students. European Journal of Dental Education. 2024;28:206–11. https://doi.org/10.1111/eje.12937.

13. Hatia A, Doldo T, Parrini S, Chisci E, Cipriani L, Montagna L, et al. Accuracy and Completeness of ChatGPT-Generated Information on Interceptive Orthodontics: A Multicenter Collaborative Study. J Clin Med. 2024;13. https://doi.org/10.3390/jcm13030735.

14. Demir GB, Süküt Y, Duran GS, Topsakal KG, Görgülü S. Enhancing systematic reviews in orthodontics: a comparative examination of GPT-3.5 and GPT-4 for generating PICO-based queries with tailored prompts and configurations. Eur J Orthod. 2024;46. https://doi.org/10.1093/ejo/cjae011.

15. Fazlollahi AM, Bakhaidar M, Alsayegh A, Yilmaz R, Winkler-Schwartz A, Mirchi N, et al. Effect of artificial intelligence tutoring vs expert instruction on learning simulated surgical skills among medical students a randomized clinical trial. JAMA Netw Open. 2022. https://doi.org/10.1001/jamanetworkopen.2021.49008.

16. El-Hakim M, Anthonappa R, Fawzy A. Artificial Intelligence in Dental Education: A Scoping Review of Applications, Challenges, and Gaps. Dent J (Basel). 2025;13:384. https://doi.org/10.3390/dj13090384.

17. Nerali JT, Chakravarthy Pishipati VK, Telang LA, Telang A. Dental Students’ Perception Towards Feedback during Clinical Training. Archives of Medicine and Health Sciences. 2021;9:62–7. https://doi.org/10.4103/amhs.amhs_212_20.

18. Aşar EM, İpek İ, Bi̇lge K. Customized GPT-4V(ision) for radiographic diagnosis: can large language model detect supernumerary teeth? BMC Oral Health. 2025;25. https://doi.org/10.1186/s12903-025-06163-3.

19. Fukuda H, Morishita M, Muraoka K, Yamaguchi S, Nakamura T, Yoshioka I, et al. Evaluating the image recognition capabilities of GPT-4V and Gemini Pro in the Japanese national dental examination. J Dent Sci. 2025;20:368–72. https://doi.org/10.1016/j.jds.2024.06.015.

20. Brin D, Sorin V, Barash Y, Konen E, Glicksberg BS, Nadkarni GN, et al. Assessing GPT-4 multimodal performance in radiological image analysis. Eur Radiol. 2025;35:1959–65. https://doi.org/10.1007/s00330-024-11035-5.

21. Uehara O, Morikawa T, Harada F, Sugiyama N, Matsuki Y, Hiraki D, et al. Performance of ChatGPT-3.5 and ChatGPT-4o in the Japanese National Dental Examination. J Dent Educ. 2024. https://doi.org/10.1002/jdd.13766.

22. Morishita M, Fukuda H, Muraoka K, Nakamura T, Hayashi M, Yoshioka I, et al. Evaluating GPT-4V’s performance in the Japanese national dental examination: A challenge explored. J Dent Sci. 2024;19:1595–600. https://doi.org/https://doi.org/10.1016/j.jds.2023.12.007.

23. Fukuda H, Morishita M, Muraoka K, Yamaguchi S, Nakamura T, Yoshioka I, et al. Evaluating the image recognition capabilities of GPT-4V and Gemini Pro in the Japanese national dental examination. J Dent Sci. 2025;20:368–72. https://doi.org/https://doi.org/10.1016/j.jds.2024.06.015.

24. Ali S, Akhlaq F, Imran AS, Kastrati Z, Daudpota SM, Moosa M. The enlightening role of explainable artificial intelligence in medical & healthcare domains: A systematic literature review. Comput Biol Med. 2023;166:107555. https://doi.org/https://doi.org/10.1016/j.compbiomed.2023.107555.

25. Çiçek FE, Ülker M, Özer M, Kıyak YS. ChatGPT versus expert feedback on clinical reasoning questions and their effect on learning: a randomized controlled trial. Postgrad Med J. 2025;101:458–63. https://doi.org/10.1093/postmj/qgae170.

26. Al-Zahrani AM. Unveiling the shadows: Beyond the hype of AI in education. Heliyon. 2024;10. https://doi.org/10.1016/j.heliyon.2024.e30696.

27. de Bruijne M. Machine learning approaches in medical image analysis: From detection to diagnosis. Med Image Anal. 2016;33:94–7. https://doi.org/https://doi.org/10.1016/j.media.2016.06.032.

28. Ribeiro MT, Singh S, Guestrin C. “Why should i trust you?” Explaining the predictions of any classifier. In: Proceedings of the ACM SIGKDD International Conference on Knowledge Discovery and Data Mining. Association for Computing Machinery; 2016. p. 1135–44. https://doi.org/10.1145/2939672.2939778.

29. Lundberg SM, Lee S-I. A Unified Approach to Interpreting Model Predictions. In: Guyon I, Luxburg U Von, Bengio S, Wallach H, Fergus R, Vishwanathan S, et al., editors. Advances in Neural Information Processing Systems. Curran Associates, Inc.; 2017.
