# Supplementary material for: Comparison of ChatGPT-4o and expert evaluation in endodontic education: a cross-sectional pilot study
Source: BMC Med Educ. 2025 Dec 1;26:12. doi: 10.1186/s12909-025-08358-2 (PMC12777446; doi:10.1186/s12909-025-08358-2)
Supplement: Supplementary file 3 — Supplementary Material 3. [file 12909_2025_8358_MOESM3_ESM.docx]

Student Opinions on the Educational Value of AI-Supported and Expert Feedback in Root Canal Shaping Practices

Instruction:

Please evaluate the following statements. For each item, mark one option between 1 (Strongly Disagree) and 5 (Strongly Agree).

1. Educational Contribution

The expert evaluation clearly showed me which points I needed to correct. [1] [2] [3] [4] [5]

The AI evaluation helped me recognize the strengths and weaknesses of my performance. [1] [2] [3] [4] [5]

Expert feedback contributed to the development of my clinical reasoning skills. [1] [2] [3] [4] [5]

AI comments were guiding for future repetitions of the practice. [1] [2] [3] [4] [5]

2. Clarity and Comprehensibility

The language and expression of the expert evaluation were clear. [1] [2] [3] [4] [5]

I did not have difficulty understanding the comments provided by AI. [1] [2] [3] [4] [5]

Both types of feedback clearly revealed the mistakes in my practice. [1] [2] [3] [4] [5]

3. Reliability and Consistency

The expert scoring seemed fair and reliable to me. [1] [2] [3] [4] [5]

The AI scoring evaluated my practice objectively. [1] [2] [3] [4] [5]

There were significant differences between the two types of feedback. [1] [2] [3] [4] [5]

4. Preference and Future Use

In the future, I would prefer AI-supported feedback in performance evaluations. [1] [2] [3] [4] [5]

The combined use of expert and AI evaluation would have been more beneficial. [1] [2] [3] [4] [5]

This experience increased my awareness about evaluation systems. [1] [2] [3] [4] [5]

5. Open-Ended Questions (optional)

What was the most striking difference between expert and AI evaluations?

In your opinion, which system was more beneficial for learning? Why?

Do you have any suggestions for improving this type of application?
